# A rare case of POEMS syndrome presenting as essential thrombocythemia

**DOI:** 10.1093/omcr/omac129

**Published:** 2022-12-16

**Authors:** Elena Angeloudi, Eleni Pappi, Aris Liakos, Maria Mainou, Efthymia Vlachaki, Eleni Bekiari

**Affiliations:** Second Department of Internal Medicine, Hippokration Hospital, Aristotle University of Thessaloniki, Thessaloniki, Greece; Second Department of Internal Medicine, Hippokration Hospital, Aristotle University of Thessaloniki, Thessaloniki, Greece; Second Department of Internal Medicine, Hippokration Hospital, Aristotle University of Thessaloniki, Thessaloniki, Greece; Second Department of Internal Medicine, Hippokration Hospital, Aristotle University of Thessaloniki, Thessaloniki, Greece; Second Department of Internal Medicine, Hippokration Hospital, Aristotle University of Thessaloniki, Thessaloniki, Greece; Second Department of Internal Medicine, Hippokration Hospital, Aristotle University of Thessaloniki, Thessaloniki, Greece

## Abstract

Polyneuropathy, organomegaly, endocrinopathy, monoclonal protein, skin changes (POEMS) is a rare paraneoplastic syndrome, and its diagnosis is based on a series of clinical and laboratory findings. We present the case of a 46-year-old woman who was previously diagnosed with essential thrombocythemia. The patient complained about dyspnea on exertion, nausea, burning of the lower limbs, weight loss, recurrent episodes of lower back pain and polymenorrhea. Physical examination revealed hyperpigmentation, livedo reticularis of the lower limbs, sclerodermoid changes and plectrodactyly. A computed tomography-guided bone biopsy revealed the presence of plasmacytoma, and based on a combination of clinical features such as polyneuropathy, a diagnosis of POEMS syndrome has been established. The diagnosis of POEMS syndrome demands a high index of suspicion, especially in cases of peripheral neuropathy, peripheral edema or organomegaly of unknown origin. Since the syndrome can be fatal, early diagnosis is pivotal for patients’ survival and quality of life.

## INTRODUCTION

Polyneuropathy, organomegaly, endocrinopathy, monoclonal protein, skin changes (POEMS) syndrome is a paraneoplastic syndrome whose acronym stands for less than half of the defining features of the disease, that is, polyneuropathy, organomegaly, endocrinopathy, monoclonal plasma cell neoplasm and skin changes [1]. The other important characteristics include papilledema, extravascular volume overload, sclerotic bone lesions, thrombocythemia, elevated vascular endothelial growth factor (VEGF) and abnormal pulmonary function [2]. Its prevalence is 0.3 per 100 000 and diagnosis is often delayed with intervening incorrect diagnoses of chronic inflammatory demyelinating polyneuropathy, myeloproliferative disorder and monoclonal gammopathy of undetermined significance. Prompt treatment directed at the underlying plasma cell clone produces dramatic responses in the majority of patients [3].

## CASE REPORT

A 46-year-old female patient was referred to the Department of Internal Medicine, Hippokration University Hospital (Thessaloniki, Greece), for assessment for potential systemic sclerosis. The patient presented with progressively worsening of acrocyanosis and Raynaud phenomenon, shortness of breath on exertion, nausea, tingling, numbness and burning of the lower limbs for a month. She also complained of weight loss (7–8 kilograms), recurrent episodes of lower back pain and polymenorrhea over the last 6 months. The patient reported a history of multiple lumbar disk hernias treated with microdiscectomy and ‘triple negative’ essential thrombocythemia (based on bone marrow findings and absence of typical genetic features) treated with acetylsalicylic acid, anagrelide and exsanguination. She also reported frequent use of analgesics for recurrent backache episodes.

**Figure 1:**
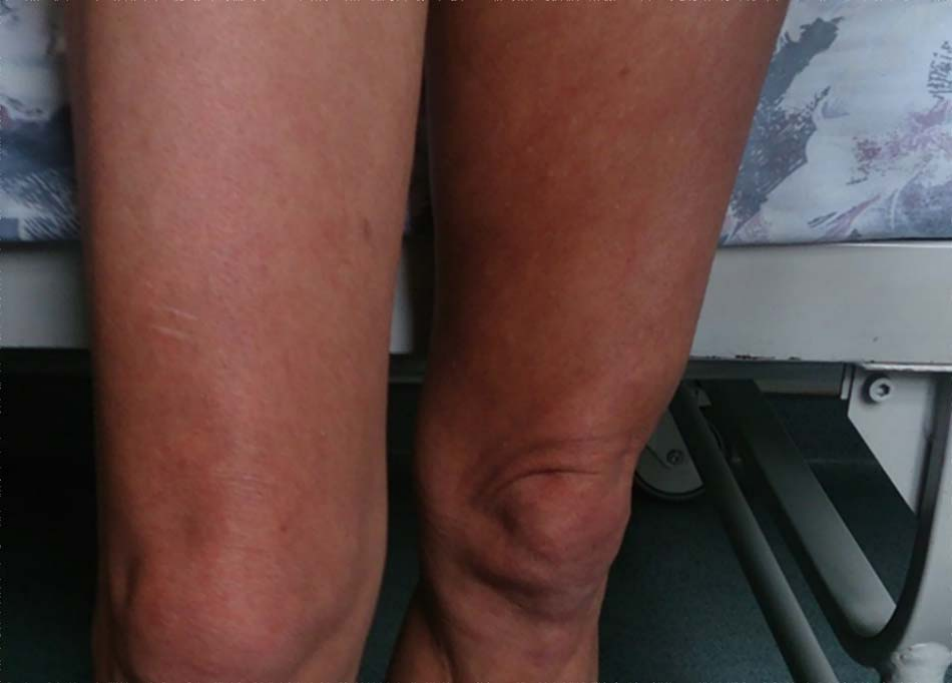
Livedo reticularis of the thighs in a patient with POEMS syndrome.

**Figure 2:**
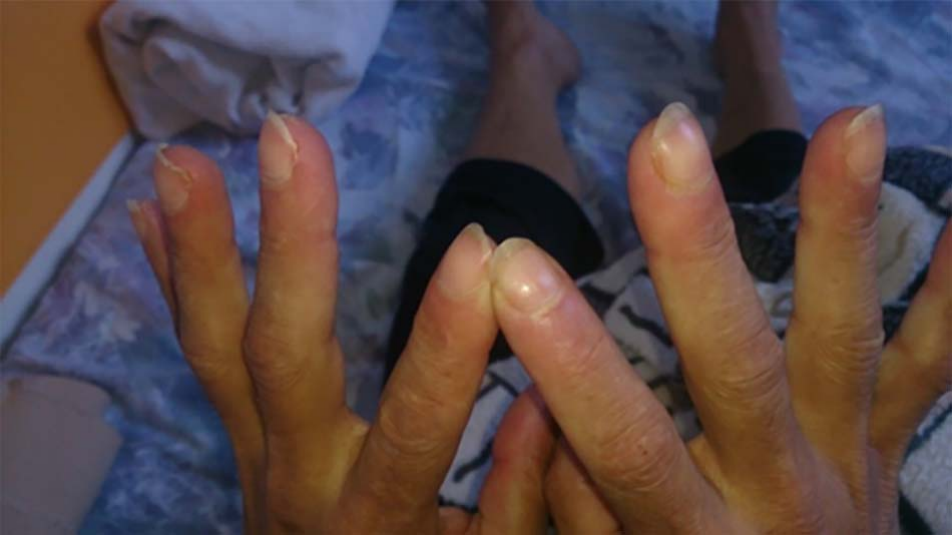
Digital clubbing in a patient with POEMS syndrome.

On admission, the patient was afebrile, non-dyspnoic, with elevated blood pressure and sinus tachycardia on the electrocardiogram. Physical examination revealed several skin abnormalities, including hyperpigmentation of the upper trunk and limbs, erythroderma, acrocyanosis, Raynaud phenomenon, leukonychia, livedo reticularis of the thighs and lower limbs, sclerodermoid changes and digital clubbing (Figs 1 and 2). Other clinical findings included bilateral jugular vein distention, lumbar oedema (anasarca) and positive right Lasegue sign. Neurological examination revealed attenuated tendon reflexes, whereas electrophysiological examination revealed a symmetric sensorimotor demyelinating polyneuropathy of the lower extremities. Fundoscopy noted bilateral papilledema. Laboratory findings on admission were unremarkable except for thrombocythemia (Table 1).

**Table 1:** Laboratory results of a patient with POEMS syndrome on admission

Laboratory results	Values
Platelet count	847 × 10^9^ cells/l
Hematocrit	42%
White cell count	9.9 × 10^9^ cells/l
Erythrocyte sedimentation rate	42 mm/h
Serum glutamic oxaloacetic transaminase	16 IU/l
Serum glutamic pyruvic transaminase	10 IU/l
Lactate dehydrogenase	229 IU/l
Albumin	4 g/dl
D-dimer	442 ng/ml
Creatinine	0.82 mg/dl
Creatine phosphokinase	344 U/l
Potassium	3.4 mEq/l
Sodium	141 mEq/l
Hepatitis B virus serological markers	Negative
Hepatitis C virus serological markers	Negative
Human immunodeficiency virus serological markers	Negative
Urine protein electrophoresis	Normal
Serum protein electrophoresis/immunofixation	Negative for monoclonal gammopathy
Immunoassay tests	Normal

**Figure 3:**
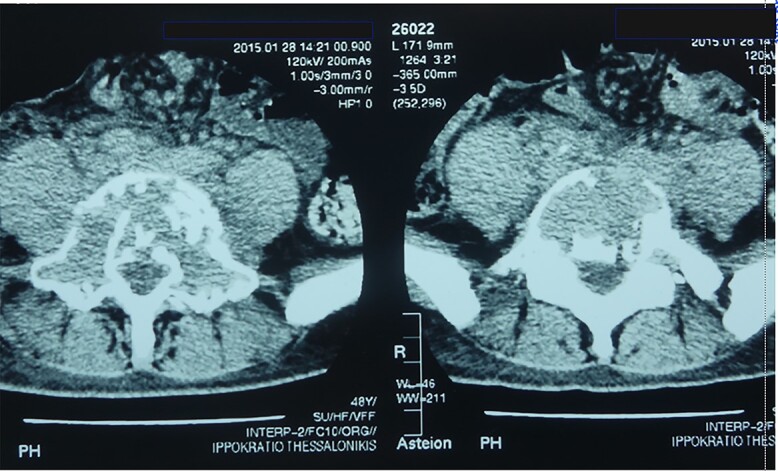
Osteosclerotic lesions of L4 and S1 vertebrae in a patient with POEMS syndrome.

Capillaroscopy revealed well-conserved capillaries, including some giant ones, and minor hemorrhages, and the findings were consistent with early scleroderma. An echocardiogram revealed pulmonary hypertension and moderate tricuspid regurgitation. Lung and abdomen computed tomography (CT) demonstrated bilateral ground-glass opacity of lower lobes and enlarged abdominal lymph nodes, respectively. Lumbar spine CT depicted degenerative and osteosclerotic lesions of L4-S1 vertebrae (Fig. 3). Magnetic resonance imaging revealed malignant lesions in L5-S1 vertebrae and pathological enrichment of quadratus lumborum and iliocostal muscles and subcutaneous loin tissue. A CT-guided bone biopsy was suggestive of plasmacytoma of bone. The patient underwent bone marrow aspiration and biopsy, which revealed a hypercellular marrow with an increase of megakaryocytes and no evidence of clonal plasma cells. An immunofixation on both serum and a 24-h urine collection was performed (and repeated) indicating absence of monoclonal gammopathy, which may be the case in a low percentage of POEMS syndrome.

The diagnosis of POEMS syndrome was established based on the presence of polyneuropathy and plasmacytoma (two mandatory criteria), sclerotic lesions of the lumbar spine (one major), thrombocythemia, thyroidal and gonadal endocrinopathy, skin alterations and bilateral papilledema (four minor) according to the 2019 update on diagnosis, risk stratification and management of POEMS syndrome [4].

Our patient had limited disease. The preferred treatment of patients with one to three isolated bone lesions is targeted radiation therapy. But, since, there was evidence of bone marrow involvement and lack of appropriate response to radiation therapy after 1 month, she was also treated with bortezomib and high-dose dexamethasone, which resulted to complete remission of the symptoms. Additional symptomatic treatment included furosemide, nifedipine and bosentan. Six months later, the patient showed a complete hematologic response and a partial radiologic response on a fluorodeoxyglucose positron emission tomography scan. Peripheral neuropathy, skin changes and symptoms of pulmonary hypertension resolved, and 30 months after diagnosis, the patient remains disease free. Our patient was then referred to a specialized myeloma center.

## DISCUSSION

The exact pathogenesis of POEMS syndrome is not completely understood, although chronic overproduction of VEGF, which increases vascular permeability, thus promoting angiogenesis, which may be an important feature of this disorder [5].

POEMS syndrome should be suspected in patients with chronic inflammatory demyelinating polyneuropathy non-responding to standard treatment or in patients with polyneuropathy combined with either paraproteinemia, thrombocythemia, oedema anasarca or papilledema. Establishment of diagnosis is based on the presence of mandatory criteria (demyelinating polyradiculoneuropathy and monoclonal plasma cell disorder) and of at least one major and one minor criterion on physical examination, imaging or laboratory evaluation [6].

A CT-guided bone biopsy was suggestive of plasmacytoma of the bone. Immunohistochemistry showed positivity for CD138, MUM1 and lamda light chains, whereas serum and urine immunofixation were negative, which may be the case in a low percentage of POEMS syndrome [7].

Our patient’s noteworthy feature was the extreme thrombocythemia for which she had been treated with anagrelide for 2 years. Thrombocythemia often constitutes an early sign of POEMS syndrome. In the Mayo Clinic POEMS series, polycythemia and thrombocythemia were observed in 54–88% of patients [5]. These conditions often lead to arterial and venous thrombosis [5]. For this reason, myeloproliferative disorders are included in the differential diagnosis of POEMS syndrome [8]. Thus, a high index of suspicion is recommended in patients with a negative molecular test for idiopathic thrombocythemia. After radiatiotherapy, high-dose dexamethasone and bortesomib, platelet count returned and remained to normal.

Half of patients with POEMS present with organomegaly typically of liver, spleen or lymph nodes [9]. Our patient presented with enlarged abdominal lymph nodes. Bilateral papilledema pointed toward diagnosis of POEMS syndrome. We also identified some typical skin manifestations such as hyperpigmentation, acrocyanosis and leukonychia. Weight loss, polymenorrhea and pulmonary hypertension also supported the diagnosis of POEMS.

Due to the fact that skin features, especially skin thickening, Raynaud’s phenomenon and hyperpigmentation, are sometimes indistinguishable from what we see in patients with systemic sclerosis, these manifestations have been referred to as scleroderma-like skin changes [10]. Besides these similarities in cutaneous appearances, abnormal activation and differentiation of B cells and generation of antibody-producing plasma cells played a role in both disorders, indicating the existence of a common pathophysiological mechanism [11]. Our patient was initially referred to our department for the assessment for potential systemic sclerosis, but since specific antibodies were negative and based on a combination of other clinical and laboratory features, a diagnosis of systemic sclerosis was excluded.

There is no standard treatment for POEMS syndrome due to the absence of available randomized controlled clinical trials. Treatment choice is generally based on whether the patient has limited or widespread sclerotic bone lesions [12]. Radiation therapy is preferred for those with up to three isolated bone lesions [6], whereas systemic therapy is recommended for patients with more disseminated disease [9]. More than half of the patients treated with radiotherapy demonstrate a significant improvement of neuropathy after several months [9]. Systemic therapies for POEMS include autologous stem cell transplantation, alkylating agents and newer immunomodulatory agents, such as thalidomide, lenalidomide and bortezomib [13]. The course of POEMS syndrome is chronic and patients survive three times longer compared with multiple myeloma. The natural history is one of progressive peripheral neuropathy until the patient is bedridden. Overall median survival was 13.7 years in the Mayo Clinic series [9], while those with clubbing or extravascular volume overload had median survivals of 2.6 and 6.6 years, respectively [12].
